# Emerging Role of Hypoxia-Inducible Factors (HIFs) in Modulating Autophagy: Perspectives on Cancer Therapy

**DOI:** 10.3390/ijms26041752

**Published:** 2025-02-19

**Authors:** Maroua Jalouli

**Affiliations:** Department of Biology, College of Science, Imam Mohammad Ibn Saud Islamic University (IMSIU), Riyadh 11623, Saudi Arabia; mejalouli@imamu.edu.sa

**Keywords:** hypoxia-inducible factors, autophagy, cancer therapy, tumor progression, drug resistance

## Abstract

Hypoxia-inducible factors (HIFs) are master regulators of cellular responses to low oxygen levels and modulate autophagy, a conserved process essential for maintaining homeostasis. Under hypoxic conditions, HIFs regulate the expression of autophagy-related genes and influence autophagic flux and cellular stress responses. Dysregulated hypoxia-induced autophagy promotes cancer cell survival, metabolism, and metastasis, thereby contributing to treatment resistance. Targeting HIF-mediated pathways or modulating autophagic processes offers the potential to improve traditional cancer therapies and overcome drug resistance. Pharmacological inhibitors of HIFs or autophagy, either alone or in combination with other treatments, may disrupt the pro-survival mechanisms within the hypoxic tumor microenvironment. Further research is needed to elucidate the intricate interplay between HIF signaling and the autophagy machinery in cancer cells. Understanding these processes could pave the way for novel therapeutic strategies to enhance treatment outcomes and combat drug resistance. This review highlights the complex relationship between HIFs and autophagy in cancer development and therapy, offering insights into how targeting these pathways may improve patient outcomes.

## 1. Introduction

Hypoxia, an important feature of the tumor microenvironment, presents an important barrier to successful cancer treatment by facilitating tumor advancement, spread, and resistance to therapy [1,2]. Hypoxia-inducible factors (HIFs) are key controllers of cellular reactions to low oxygen levels and are crucial for facilitating these adaptive responses [3]. Autophagy has been identified as a crucial mechanism for cell survival and adaptability in hypoxic environments among the various cellular processes regulated by HIFs [4]. HIFs and autophagy are both triggered in the reaction to hypoxia, a condition characterized by reduced quantities of oxygen in cells [5]. HIFs are a type of transcription factor that control how cells respond to low oxygen levels [3]. They play a crucial role in regulating processes, such as autophagy, angiogenesis, and metabolic adaptation [4]. Autophagy is crucial for the survival and adaptation of cancer cells in demanding situations, such as hypoxia and the tumor microenvironment [6]. The precise biochemical mechanism behind the impact of HIFs and autophagy on cancer cell proliferation, progression, and resistance to cancer treatment remains unclear.

Autophagy is a cellular process in which intracellular components are broken down and recycled through self-digestion [7]. Targeting the HIF-mediated autophagy system is a viable therapeutic option due to its crucial involvement in cancer growth and resistance to therapy. Various strategies have been proposed to regulate HIF-induced autophagy, including the use of drugs to inhibit HIF signaling, the modulation of autophagy regulators, and combination therapies that simultaneously target both pathways [8]. Compounds that disrupt HIF signaling pathways, including HIF-1α inhibitors and prolyl hydroxylase inhibitors, have demonstrated potential in preclinical research by effectively suppressing HIF-mediated autophagy and improving the effectiveness of standard cancer treatments [9]. Moreover, researchers are also investigating combination therapies that involve the use of autophagy inhibitors along with conventional chemotherapeutic medicines or targeted therapies. The objective is to overcome resistance to treatment and improve the outcomes for patients by inducing autophagy through HIFs. Increasing evidence indicates that HIFs have a pivotal function in controlling autophagy in cancer cells when exposed to low oxygen levels [10]. HIF-1α has been demonstrated to increase the transcription of various autophagy-related genes, such as BNIP3 (BCL2/adenovirus E1B 19kDa protein-interacting protein 3) and BNIP3L/NIX (BCL2/adenovirus E1B 19kDa interacting-protein 3-like) [11]. These genes play a role in the initiation of mitophagy, a specific type of autophagy that aims to eliminate impaired mitochondria. In addition, it has been shown that HIF-1α increases the expression of genes that encode lysosomal proteins, which aids in the merging of autophagosomes with lysosomes and enhances the flow of autophagy [4]. The findings indicate that HIF-1α stimulates autophagy in cancer cells as a means of survival to cope with hypoxic stress circumstances. In contrast, other research studies have proposed that HIF-1α has a role in suppressing autophagy in specific circumstances [4]. For instance, it has been documented that HIF-1α increases the transcriptional activity of REDD1, a protein involved in development, as well as DNA damage responses [12]. REDD1, in turn, suppresses mTORC1, a crucial suppressor of autophagy [13]. HIF-1α can inhibit autophagy in nutrient-rich environments or in response to growth factor stimulation by activating mTORC1 [14]. Nevertheless, the specific function of HIF-1α in controlling autophagy can differ based on the cellular circumstances and environmental signals found in the tumor microenvironment [15]. In this review, new insights are provided to elucidate the role that HIF-mediated autophagy plays in the development and treatment of cancer. Additionally, attention is drawn to the ways in which HIF-mediated autophagy may alter the ability to overcome treatment resistance and improve patient outcomes in cancer.

## 2. Important Function of HIFs in the Progression of Cancer Cells

**Figure 1:**
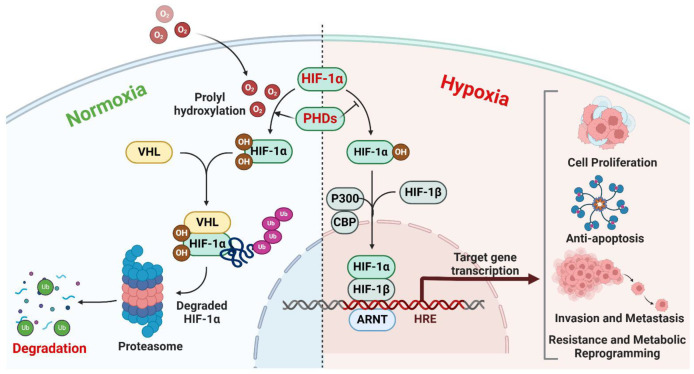
Molecular mechanism of hypoxia-inducible factors (HIFs) in normoxic and hypoxic conditions. Under normoxic conditions, prolyl hydroxylase domain-containing enzymes (PHDs) hydroxylate HIF-α subunits, facilitating their recognition and degradation by the Von Hippel–Lindau tumor suppressor protein (VHL) via proteasomal polyubiquitination. In hypoxia, PHDs and FIH lose the ability to hydroxylate HIF-α, allowing HIF-α to translocate into the nucleus. There, HIF-α dimerizes with HIF-1β and recruits the coactivators p300 and CBP. The resulting complex binds to hypoxia-response elements (HREs) in target gene promoters, activating genes involved in processes, such as cell proliferation, anti-apoptosis, invasion, metastasis, resistance to therapy, and metabolic reprogramming.

HIFs are transcription factors that consist of an oxygen-sensitive α subunit (HIF-1α, HIF-2α, or HIF-3α), in addition to a β subunit (HIF-1β/ARNT) that is expressed continuously [16,17]. Under normal oxygen conditions, prolyl hydroxylase domain proteins (PHDs) hydroxylate HIF-α subunits, which leads to their ubiquitination and subsequent destruction by the proteasome [18]. Nevertheless, in situations with low oxygen levels, the process of oxygen-dependent hydroxylation is hindered, resulting in the stability of HIF-α subunits and their movement into the nucleus [19]. HIF heterodimers bind to hypoxia-response elements (HREs) in target genes, which triggers the initiation of transcriptional programs that promote cell survival, angiogenesis, metabolic adaptability, and metastasis [15] (Figure 1). An important role of HIFs in the advancement of cancer is the stimulation of angiogenesis, which is the process of creating new blood vessels from existing ones [8]. However, the release of vascular endothelial growth factors (VEGFs), in addition to other pro-angiogenic factors, driven by HIFs, promotes the proliferation of endothelial cells and increases the permeability of blood vessels [20]. This process facilitates the formation of blood vessels in tumors and enhances the delivery of nutrients. In addition, HIFs regulate metabolic changes in cancer cells, favoring glycolysis instead of oxidative phosphorylation, even in the presence of sufficient oxygen [21]. This metabolic shift is commonly known as the Warburg effect [22]. It not only supports the growth of tumors in areas with low oxygen levels but also provides them with a competitive advantage in terms of cell proliferation and resistance to chemotherapy. Tumor cells utilize glycolysis for ATP production and survival, particularly in hypoxic environments [23]. This metabolic adaptation ensures a continuous energy supply and confers resistance to chemotherapy via multiple mechanisms. Glycolysis reduces mitochondrial oxidative phosphorylation, resulting in lower ROS production [24]. The induction of apoptosis by numerous chemotherapeutic agents through elevated ROS levels results in diminished efficacy due to this metabolic shift. The glycolytic pathway generates lactate, which plays a role in extracellular acidification [25]. The acidic microenvironment hinders the uptake and effectiveness of chemotherapeutic agents, many of which are sensitive to pH levels. The glycolytic shift activates pro-survival pathways, including HIF-1α signaling, which promotes the expression of anti-apoptotic proteins and enhances drug resistance [26]. Glycolysis generates intermediates essential for nucleotide biosynthesis, thereby enhancing DNA repair mechanisms and diminishing the efficacy of DNA-damaging chemotherapeutic agents [27].

HIFs have a crucial function in promoting epithelial–mesenchymal transition (EMT), which is a process wherein cells lose their epithelial characteristics and gain mesenchymal qualities [28]. The process of EMT grants cancer cells with an increased ability to move, invade surrounding tissues, and resist apoptosis [29]. This enables them to locally invade nearby tissues and spread to distant sites (metastasis). The activation of EMT transcription factors, including Slug, Snail, and Twist, triggered by HIFs, coordinates the restructuring of the cytoskeleton, degradation of the extracellular matrix, and changes in cell–cell adhesion molecules [30]. This process facilitates the spread of cancer cells from the original tumor to distant locations. An increasing amount of data indicates that HIFs play a role in immune evasion by altering the tumor microenvironment [31]. HIF activation leads to an increase in the expression of immunosuppressive molecules, such as programmed death-ligand 1 (PD-L1), indoleamine 2,3-dioxygenase (IDO), and regulatory T cells (Tregs) [32]. This suppresses the function of effector T cells and increases immunological tolerance [33]. In addition, HIFs drive the polarization of tumor-associated macrophages (TAMs) towards an M2-like phenotype [34]. This phenotype further enhances tumor development, angiogenesis, and tissue remodeling [35]. HIFs play a role in creating a suppressive environment in the tumor microenvironment, which allows the tumor to evade the immune system and promotes its growth [36].

## 3. Role of Hypoxia-Inducible Factors in Modulating Autophagy and Maintaining Cellular Homeostasis

It has been established that autophagy is a method for intracellular self-degradation. This mechanism is responsible for the termination of aggregated misfolded proteins and defective cytoplasmic organelles by means of fusion with lysosomes and double-membrane autophagosomes. This process is necessary for the maintenance of cellular homeostasis [37]. In most cases, the process of autophagy is started by isolating pre-autophagosome structures, which are referred to as phagophore assembly sites (PASs) [38,39]. PI3K, which is associated with the ER, has a fundamental function in the development of PASs [40]. During the induction of autophagy, the formation of phagophores is facilitated by key proteins, including autophagy-activating kinase 1 (ULK1), the mammalian target of rapamycin (mTOR), and AMP-activated protein kinase (AMPK). Unc-51 also plays a crucial role in this process [41]. Nevertheless, the VPS34/UVRAG/Beclin-1/AMBRA1 complex contributes to the generation of phagophores, which are formed through membrane elongation and the subsequent development of autophagosomes [42,43]. Through the connection of the ESCRT/SNARE/Rab7 protein complex, lysosomes can link to the mature autophagosomes, which ultimately leads to the production of autolysosomes in the cell [44,45].

**Figure 2:**
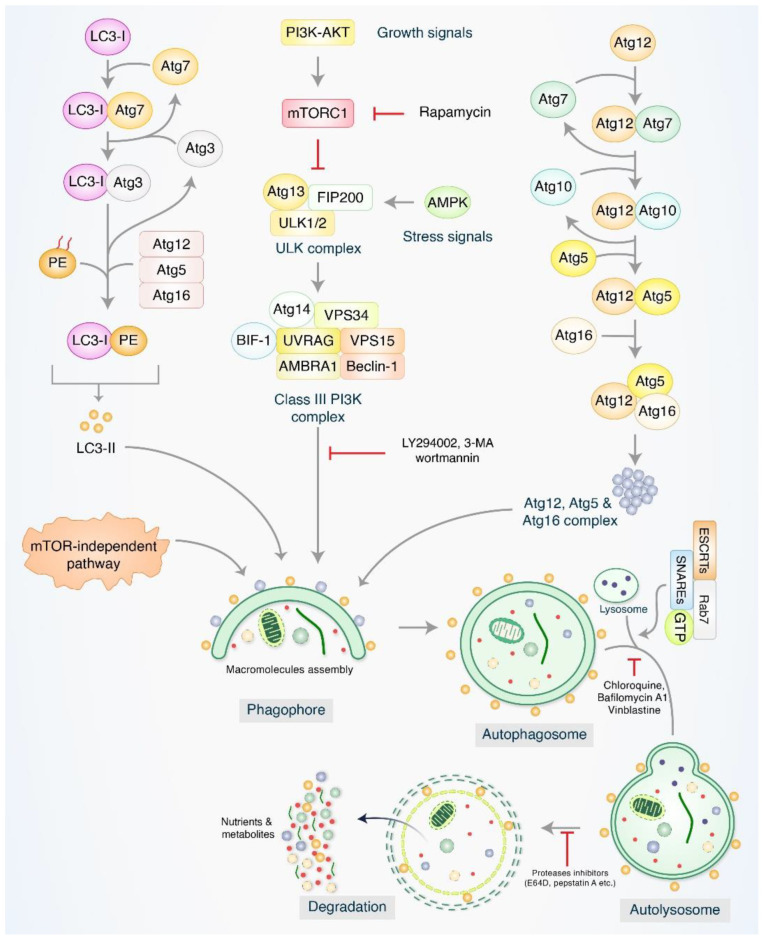
Biological function of the autophagy pathway, as well as its molecular mechanism. Through the collaborative efforts of several proteins, the process of autophagy is kicked off by the development of a pre-autophagosome structure. Through the interaction of the ULK1/VPS34/Beclin-1 complex, PI3K–AKT and mTOR are induced to initiate the assembly of pre-autophagosomes. In addition, the Atg5/Atg12/Atg16 and Atg12/Atg5/LC3 complexes are engaged in the process of phagophore nucleation and the accumulation of macromolecules that have been elongated. These complexes also bind to autophagosomes. Mature autophagosomes are bound to lysosomes with the assistance of the ESCRT/SNARE/Rab7 protein complex, which ultimately leads to the production of autolysosomes. Finally, acid hydrolases eliminate autolysosomes, which leads to the release of recycling metabolites, in addition to nutrients.

On the other hand, autolysosomes that include misfolded or aggregated proteins are destroyed by acid hydrolases. These autolysosomes supply recycling metabolites and nutrients, which are necessary for the maintenance of intracellular homeostasis (Figure 2). The autophagy process is accountable for the regulation of cancer cell destiny and the formation of cancer cells [46]. There is a growing body of research suggesting that autophagy could be the determining factor of whether cancer cells are fostered or repressed under predetermined circumstances [47].

**Figure 3:**
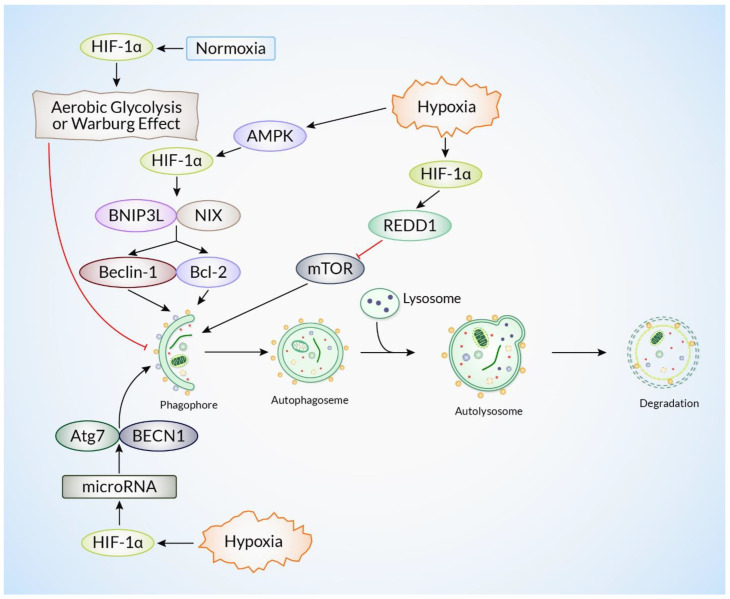
The role of HIF-1α in hypoxia-induced autophagy. Hypoxic stress stabilizes and results in the accumulation of HIF-1α, triggering hypoxia-induced autophagy. HIF-1α upregulates NIX (BNIP3L) and BNIP3, disrupting the Beclin-1/Bcl-2 interaction and freeing Beclin-1 to activate autophagy initiation. This cascade recruits ATG5 and LC3, promoting autophagosome formation. Additionally, HIF-1α enhances autophagy by upregulating REDD1, which suppresses mTOR. HIF-1α also activates specific microRNAs and Atg proteins, further enhancing autophagy initiation. This process facilitates the clearance of damaged organelles and maintains cellular homeostasis under hypoxic conditions.

Recent findings indicate a complex relationship between HIFs and autophagy, where HIFs can have both beneficial and detrimental effects on autophagy depending on the specific circumstances and the duration of exposure to low oxygen levels [3,48]. BNIP3L and NIX genes facilitate the initiation of autophagy by disrupting the interaction between Beclin-1 and Bcl-2, thereby freeing Beclin-1 to activate autophagosome formation [49]. In addition, HIF-1α can activate autophagy by promoting the production of Regulated in Development and DNA Damage Response 1 (REDD1), which suppresses the mechanistic target of rapamycin complex 1 (mTORC1) pathway, a factor that limits autophagy [50]. Extended exposure to hypoxia can cause the inhibition of autophagy through different pathways that involve HIFs [4]. HIF-1α has been found to increase the production of miR-210, a microRNA that regulates autophagy genes, such as ATG7 and BECN1 [51], leading to the inhibition of autophagy induction (Figure 3). In addition, HIF-1α can impede autophagy by stimulating the production of p62/SQSTM1 (sequestosome 1), a protein that acts as a receptor for selective autophagy and participates in the breakdown of ubiquitinated proteins through the autophagy–lysosomal pathway [52]. Moreover, HIF-1α might indirectly hinder autophagy by stimulating aerobic glycolysis (known as the Warburg effect), a process that produces ATP through glycolysis even when oxygen is available [53]. This, in turn, decreases the cellular requirement for autophagy-driven energy generation.

**Figure 4:**
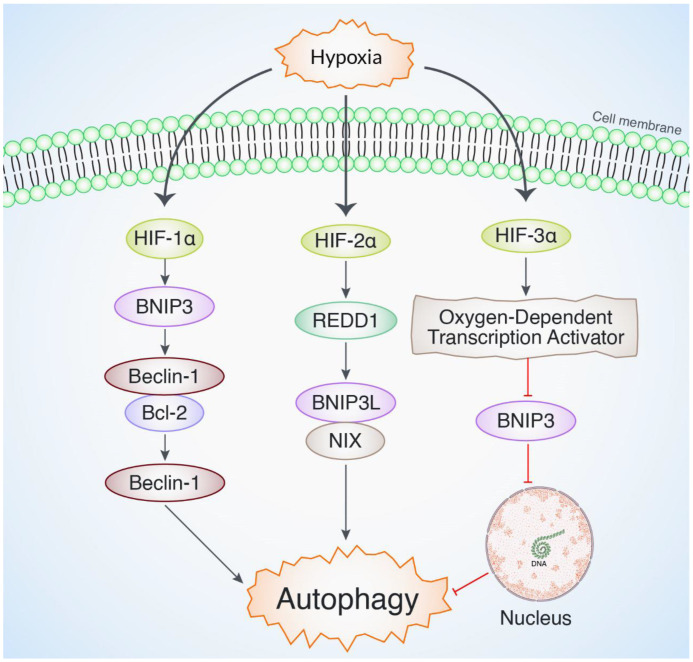
HIF isoforms, including HIF-2α and HIF-3α, also regulate autophagy in low-oxygen environments. They alter genes differently and use different approaches. Research suggests that HIF-2α, like HIF-1α, can promote autophagy by increasing the synthesis of REDD1 and BNIP3L/NIX. HIF-3α negatively impacts autophagy by inhibiting BNIP3 synthesis. This suppresses mitophagy and promotes mitochondrial growth in low oxygen conditions.

Other HIF isoforms, including HIF-2α and HIF-3α, also regulate autophagy in low-oxygen environments [43,49]. They alter genes differently and use different approaches. Research suggests that HIF-2α, like HIF-1α, can promote autophagy by increasing the synthesis of REDD1 and BNIP3L/NIX [54]. On the other hand, HIF-3α has been found to have a detrimental effect on autophagy by blocking the production of BNIP3. This leads to the suppression of mitophagy (the selective removal of damaged mitochondria) and promotes the creation of new mitochondria in low oxygen situations [55] (Figure 4). Further research is needed to understand the exact molecular mechanisms that control the interaction between HIFs and autophagy. This review aims to identify new targets for the development of new treatment approaches for disorders related to hypoxia and the dysregulation of autophagy. Moreover, investigating the therapeutic capacity of pharmacological agents that can modify HIFs and autophagy in both preclinical and clinical environments has potential for creating targeted treatments with enhanced effectiveness and safety characteristics.

## 4. Recent Drug Targets for HIF-Mediated Autophagy Regulation in Cancer

Researchers have recently discovered multiple therapeutic targets in the HIF-mediated autophagy pathway for the treatment of cancer. Promising outcomes have been shown in preclinical investigations using small-molecule inhibitors that target HIFs or crucial elements of the autophagy mechanism. Furthermore, the use of combination therapies that combine HIFs or autophagy inhibitors with traditional chemotherapeutic agents or targeted therapies has shown to have a synergistic impact on inhibiting tumor growth and overcoming drug resistance [52]. Advances in nanotechnology have facilitated the creation of precise drug delivery methods to improve effectiveness and minimize the adverse effects of HIFs or autophagy inhibitors in cancer treatment [52]. These novel methodologies have significant potential for enhancing patient outcomes and promoting the progress of precision medicine in the field of oncology. To summarize, focusing on the regulation of autophagy through HIF-mediated pathways shows potential as a technique for creating new and more effective cancer treatments that are more selective.

### 4.1. Natural Products for HIF-Mediated Autophagy Modulation in Cancer

Tumor growth and metastasis are complex processes regulated by the tumor microenvironment, with a critical component being the regulation of hypoxia-inducible factor (HIF)-mediated autophagy. Natural chemicals have arisen as effective modulators of this process, presenting a viable strategy for cancer treatment [56,57]. Various natural compounds have the capacity to regulate autophagy through HIF modulation (Table 1), presenting encouraging possibilities for tumor and cancer treatment.

Resveratrol, a polyphenolic molecule found in grapes, is recognized for its anti-cancer potential. In vitro investigations utilizing MCF-7 breast cancer cells have shown that resveratrol suppresses HIF-1α expression, which is essential for cancer cells to adapt to hypoxic environments. Furthermore, resveratrol stimulates autophagy via the AMP-activated protein kinase (AMPK)–mechanistic target of rapamycin (mTOR) pathway. This dual mechanism indicates that resveratrol may inhibit tumor proliferation while promoting the apoptosis of cancer cells in hypoxic environments [58]. Curcumin, a bioactive constituent of turmeric, has attracted interest in its capacity to impede HIF-1α stabilization in glioblastoma cells (U87MG). Curcumin activates autophagy primarily by inhibiting the PI3K/Akt/mTOR signaling pathway, which is crucial for cellular development and survival. Its capacity to regulate HIFs and autophagy in glioblastoma highlights its therapeutic promise in hypoxic malignancies [59]. Epigallocatechin gallate (EGCG), the primary catechin in green tea, has demonstrated the ability to diminish HIF-1α activity in lung cancer, both in vitro and in vivo. In murine models, EGCG induces autophagy through AMPK activation, which aids the anti-cancer properties of EGCG by inhibiting tumor growth via modulation of the HIF pathway and promoting the clearance of damaged cellular components through autophagy [60]. Berberine, an alkaloid present in various plants, demonstrates anti-cancer properties by inhibiting HIF-1α signaling in colorectal cancer cells (HCT116). This chemical initiates autophagy by causing oxidative stress, which activates autophagic pathways via ROS. Berberine’s capacity to regulate both HIFs and autophagy underscores its promise as a therapeutic agent in colorectal cancer [61]. Quercetin, found in numerous fruits and vegetables, has demonstrated the ability to reduce HIF-1α protein levels in pancreatic cancer. In vitro and in vivo studies indicate that quercetin stimulates autophagy by inhibiting the Akt/mTOR pathway, elucidating a mechanism by which quercetin can restrict tumor development and enhance cancer cell viability under hypoxic settings [62]. Genistein, has been recognized as a regulator of HIF-1α in prostate cancer cells (PC-3). Genistein diminishes HIF-1α expression and stimulates autophagy via the JNK pathway to facilitate cellular death under hypoxic conditions. This indicates that genistein’s effect on both HIFs and autophagy could be advantageous in addressing the shortcomings of existing prostate cancer therapies [63]. Apigenin, has been found to exhibit anti-cancer effects in liver cancer models. It suppresses HIF-1α transcription and induces autophagy through mTOR inhibition. Apigenin’s combined regulation of HIFs and autophagy presents a promising strategy for liver cancer treatment, particularly in hypoxic tumor environments [64]. Honokiol, a chemical extracted from the bark of Magnolia trees, has been shown to diminish HIF-1α expression and stimulate autophagy in glioblastoma, both in vitro and in vivo. This chemical operates through the AMPK pathway, underscoring its potential as an anti-cancer drug, especially in glioblastoma, a malignancy recognized for its hypoxic areas [65]. Withaferin A, a steroidal lactone derived from *Withania somnifera* (ashwagandha), has demonstrated the ability to decrease HIF-1α production in breast cancer cells (MDA-MB-231). It also stimulates autophagy by stimulating ROS generation and endoplasmic reticulum (ER) stress pathways. Withaferin A’s capacity to affect both HIFs and autophagy underscores its promise as a therapeutic drug in breast cancer care [66]. Capsaicin, the active component that imparts spiciness to chili peppers, diminishes HIF-1α transcription in lung adenocarcinoma (A549 cells). Capsaicin stimulates autophagy via the AMPK pathway, thus augmenting the removal of defective organelles and proteins, which may improve therapeutic outcomes for patients with lung cancer [67]. Kaempferol, has demonstrated the ability to alter HIF-1α signaling in ovarian cancer. In vivo investigations have shown that kaempferol suppresses HIF-1α expression and promotes autophagy by inhibiting the Akt/mTOR pathway. These actions render kaempferol an attractive choice for ovarian cancer treatment in hypoxic situations [68]. Rhein, a chemical extracted from the rhubarb plant, inhibits HIF-1α expression in hepatocellular carcinoma (HepG2 cells). Its capacity to activate autophagy is facilitated through ROS-dependent pathways, indicating that rhein can affect tumor growth in hepatocellular carcinoma by targeting both HIFs and autophagy [69]. Luteolin, suppresses HIF-1α transcription in colon carcinoma. Luteolin promotes autophagy in mouse models by inhibiting the PI3K/Akt signaling pathway. Luteolin’s capacity to regulate both HIFs and autophagy offers a novel strategy for restricting tumor proliferation in colon cancer [70]. Baicalin, derived from *Scutellaria baicalensis*, has demonstrated the ability to diminish HIF-1α expression in esophageal cancer (ECA109 cells). It induces autophagy through AMPK/mTOR signaling, hence enhancing its anti-cancer properties, especially under hypoxic conditions in esophageal cancers [71]. Diallyl trisulfide, a garlic-derived chemical, suppresses HIF-1α transcription in stomach cancer. Its capacity to stimulate autophagy is associated with the suppression of mTOR signaling, rendering it a promising candidate for improving the effectiveness of therapies aimed at gastric cancer [72].

The regulation of HIF-mediated autophagy by natural substances is fascinating, although numerous limitations must be addressed. Several research studies, such as those examining diosmetin and salidroside, utilized cell culture models that may inadequately reflect the tumor microenvironment. Moreover, the effectiveness of these substances in clinical environments is largely unexamined, requiring thorough in vivo and clinical investigations. Moreover, several substances, such as caffeic acid and berberine, exhibit heterogeneity in their effects, contingent upon the dosage and experimental settings. A systematic methodology for assessing these substances is crucial for converting preclinical results into medicinal approaches. Additional study is necessary to address the current limitations and substantiate these findings in therapeutically pertinent environments. Integrating natural substances into therapeutic frameworks may facilitate the development of more effective and less harmful cancer treatments.

**Table 1:** Natural compounds that modulate HIF-mediated autophagy regulation in different cancers.

Natural Compound	Tumor Type	Experimental System	Effect on HIFs	Effect on Autophagy	References
Resveratrol	Breast cancer	In vitro (MCF-7 cells)	Downregulates HIF-1α expression	Activates autophagy via AMPK–mTOR pathway	[58,73]
Curcumin	Glioblastoma	In vitro (U87MG cells)	Inhibits HIF-1α stabilization	Induces autophagy by inhibiting PI3K/Akt/mTOR	[59,74]
Epigallocatechin gallate	Lung cancer	In vivo (mouse model)	Reduces HIF-1α activity	Promotes autophagy through AMPK activation	[60,75]
Berberine	Colorectal cancer	In vitro (HCT116 cells)	Suppresses HIF-1α signaling	Induces autophagy through ROS-mediated pathway	[61,76]
Quercetin	Pancreatic cancer	In vivo and in vitro	Downregulates HIF-1α protein levels	Activates autophagy through inhibition of Akt/mTOR	[62,77]
Genistein	Prostate cancer	In vitro (PC-3 cells)	Decreases HIF-1α expression	Promotes autophagy via JNK and Beclin-1 activation	[63,78]
Apigenin	Liver cancer	In vivo (rat model)	Inhibits HIF-1α transcription	Induces autophagy by inhibiting mTOR	[64,79]
Honokiol	Glioblastoma	In vivo and in vitro	Downregulates HIF-1α and VEGF expression	Triggers autophagy through AMPK activation	[65,80]
Withaferin A	Breast cancer	In vitro (MDA-MB-231)	Inhibits HIF-1α expression	Induces autophagy through ROS and ER stress	[66,81]
Capsaicin	Lung adenocarcinoma	In vitro (A549 cells)	Reduces HIF-1α transcription	Activates autophagy via AMPK signaling	[67,82]
Kaempferol	Ovarian cancer	In vivo (mouse model)	Inhibits HIF-1α signaling	Enhances autophagy via Akt/mTOR inhibition	[68,83]
Rhein	Hepatocellular carcinoma	In vitro (HepG2 cells)	Suppresses HIF-1α protein expression	Promotes autophagy through ROS-dependent pathways	[69,84]
Luteolin	Colon cancer	In vivo (mouse model)	Inhibits HIF-1α transcription	Induces autophagy by blocking PI3K/Akt signaling	[70,85]
Baicalin	Esophageal cancer	In vitro (ECA109 cells)	Downregulates HIF-1α expression	Activates autophagy via AMPK–mTOR signaling	[71,86]
Diallyl trisulfide	Gastric cancer	In vivo (mouse model)	Inhibits HIF-1α transcription	Triggers autophagy via inhibition of mTOR signaling	[72,87]

### 4.2. Synthetic Drugs for HIF-Mediated Autophagy Regulation in Cancer

Synthetic chemicals can influence HIF-mediated autophagy regulation in various cancer types. These metabolites modulate HIF-1α activity, thereby regulating autophagy pathways. Several synthetic chemicals effectively suppress autophagy by modulating HIF-1α activity, as summarized in Table 2.

#### 4.2.1. HIF-1α Inhibition and Autophagy Suppression

Numerous synthetic chemicals effectively inhibit autophagy through the suppression of HIF-1α activity. Digoxin, a recognized cardiac glycoside, diminishes autophagy by downregulating HIF-1α expression in breast cancer cells at concentrations ranging from 50 to 100 nM [88]. PX-478, a potent HIF-1α transcription inhibitor, similarly suppresses autophagy in glioblastoma at concentrations ranging from 10 to 50 µM [89]. 2-Methoxyestradiol (2-ME) destabilizes HIF-1α, leading to a reduction in autophagy induction in pancreatic cancer at concentrations of 1–10 µM [90]. Acriflavine inhibits HIF-1 dimerization, disrupts the HIF-1α/β interaction, and effectively suppresses autophagy in prostate cancer cells at concentrations of 5–25 µM. Bortezomib, a proteasome inhibitor, promotes the degradation of HIF-1α, resulting in the suppression of autophagy in multiple myeloma at concentrations ranging from 10 to 50 nM [91]. YC-1 (1–50 µM) similarly inhibits the transcriptional activity of HIF-1α, leading to a reduction in autophagy within colon cancer cells [92].

#### 4.2.2. Targeting Hypoxia-Induced Autophagy

Specific synthetic chemicals target autophagy induced by hypoxia. Tirapazamine, a hypoxia-activated prodrug, inhibits hypoxia-induced autophagy in head and neck cancer cells at concentrations ranging from 10 to 100 µM [93]. LW6 (1–10 µM) facilitates the degradation of HIF-1α and suppresses autophagy in lung cancer cells [94]. Vorinostat (SAHA), an HDAC inhibitor, reduces HIF-1α expression, thereby inhibiting autophagy in glioblastoma at concentrations of 1–10 µM [95].

##### Chemotherapeutics Affecting HIF-Mediated Autophagy

Common chemotherapeutic agents regulate autophagy through the modulation of HIF-1α. Doxorubicin, a commonly utilized anticancer agent, inhibits HIF-1α activity and diminishes autophagic survival in breast cancer at concentrations ranging from 0.1 to 5 µM [96]. Camptothecin, an inhibitor of topoisomerase I, suppresses the stabilization of HIF-1α and autophagy in colon cancer cells at concentrations ranging from 10 to 100 nM [97]. Temsirolimus, an mTOR inhibitor, obstructs mTOR signaling, consequently augmenting HIF-mediated autophagy in renal cell carcinoma at concentrations of 10–50 nM [98]. Sorafenib, a multi-kinase inhibitor, reduces autophagy in liver cancer through the inhibition of HIF-1α at concentrations ranging from 1 to 10 µM [99].

##### Emerging Small Molecules and HIF-1α Modulation

Recent advancements in targeted therapy have resulted in the creation of new HIF-1α modulators. EZN-2208, an antisense oligonucleotide directed against HIF-1α, significantly decreases its expression and suppresses autophagy in pancreatic cancer at concentrations ranging from 10 to 100 nM [100]. CH5132799, a PI3K/mTOR inhibitor, inhibits HIF-mediated autophagy in breast cancer cells at concentrations of 1–10 µM [101].

**Table 2:** Synthetic chemicals that affect HIF-mediated autophagy and their implications for cancer therapy.

**Synthetic Chemical**	**Target Pathway**	**Cancer Type**	**Concentration/** **Dose**	**Mechanism of Action**	**Ref.**
Digoxin	HIF-1α inhibitor	Breast cancer	50–100 nM	Inhibits HIF-1α expression to reduce autophagy	[88]
PX-478	HIF-1α inhibitor	Glioblastoma	10–50 µM	Blocks HIF-1α transcription to suppress autophagy	[89]
2-Methoxyestradiol (2-ME)	HIF-1α inhibitor	Pancreatic cancer	1–10 µM	Destabilizes HIF-1α to reduce autophagy induction	[90]
Bortezomib	Proteasome inhibitor	Multiple myeloma	10–50 nM	Increases HIF-1α degradation to reduce autophagic survival	[91]
YC-1	HIF-1α inhibitor	Colon cancer	1–50 µM	Inhibits HIF-1α transcriptional activity to reduce autophagy	[92]
Tirapazamine	Hypoxia-activated prodrug	Head and Neck cancer	10–100 µM	Disrupts hypoxia-induced autophagy by suppressing HIF-1α	[93]
LW6	HIF-1α degradation inducer	Lung cancer	1–10 µM	Promotes HIF-1α proteasomal degradation and reduces autophagy	[94]
Vorinostat (SAHA)	HDAC inhibitor	Glioblastoma	1–10 µM	Inhibits HIF-1α expression and blocks hypoxia-induced autophagy	[95]
Doxorubicin	Topoisomerase II inhibitor	Breast cancer	0.1–5 µM	Suppresses HIF-1α activity and reduces autophagic cell survival	[96]
Camptothecin	DNA topoisomerase I inhibitor	Colon cancer	10–100 nM	Inhibits HIF-1α stabilization, thereby affecting autophagy	[97]
Temsirolimus	mTOR inhibitor	Renal cell carcinoma	10–50 nM	Blocks mTOR and enhances HIF-mediated autophagy	[98]
Sorafenib	Multi-kinase inhibitor	Liver cancer	1–10 µM	Inhibits HIF-1α and downregulates autophagy in hypoxic environment	[99]
EZN-2208	HIF-1α antisense oligonucleotide	Pancreatic cancer	10–100 nM	Reduces HIF-1α expression and inhibits autophagy	[100]
CH5132799	PI3K/mTOR inhibitor	Breast cancer	1–10 µM	Suppresses HIF-mediated autophagy under hypoxia	[101]

### 4.3. MicroRNAs for HIF-Mediated Autophagy Regulation in Cancer

MicroRNAs (miRNAs) are small, non-coding RNAs that modulate gene expression by binding to the 3′-untranslated region (UTR) of target mRNAs, resulting in their damage or the inhibition of translation. MicroRNAs significantly regulate various cellular processes in cancer, including autophagy and responses mediated by hypoxia-inducible factors (HIFs). A recent study has emphasized the role of some miRNAs in regulating HIF-mediated autophagy in cancer [10]. MicroRNAs function as regulators, exerting precise control over autophagic activity in reaction to hypoxic environments [102]. They affect tumor cell survival, metabolism, and responsiveness to therapy by specifically targeting important elements of the HIF signaling pathway or autophagic machinery [103]. An area of research with great potential is the advancement of miRNA-based treatments to regulate HIF-mediated autophagy in cancer [104]. Researchers have sought to manipulate the regulatory capability of miRNAs to disturb the intricate equilibrium of autophagy in tumor cells [105]. This disruption results in a reduced lifespan for tumor cells and increased vulnerability to therapy. Table 3 presents a summary of microRNAs that play a role in regulating HIF-mediated autophagy in cancer.

The expression of microRNA-21 increases the levels of HIF-1α through the promotion of autophagy in various cancer cell types [106]. microRNA-210 is a small RNA molecule directly targeting HIF-1α mRNA in hypoxic cells to enhance autophagy [107]. The translation of HIF-1α is inhibited in different cells by microRNA-155, leading to the suppression of autophagy [108]. The stability of HIF-1α in breast cancer cells is regulated by microRNA-31, leading to enhanced autophagy [109]. The expression of HIF-1α is modulated by microRNA-29a, leading to the suppression of autophagy in lung cancer cells [110]. MicroRNA-23a inhibits the process of autophagy in renal cell cancer by suppressing the HIF-1α pathway [111]. MicroRNA-519c inhibits autophagy in gastric cancer cells by specifically targeting HIF-1α [112]. MicroRNA-20a reduces HIF-1α and hence promotes autophagy in colon cancer cells [113]. The expression of HIF-1α is regulated by microRNA-424, leading to the suppression of autophagy in breast cancer cells [114]. The activity of HIF-1α is modified by microRNA-27a to increase autophagy in pancreatic cancer [115]. Furthermore, the identification of circulating miRNAs as potential biomarkers provides non-invasive methods for cancer detection, the prediction of outcomes, and tracking the effectiveness of treatments. Clarifying the function of miRNAs in regulating HIF-mediated autophagy will offer significant knowledge about cancer biology and prospective therapeutic approaches that could revolutionize cancer treatment.

**Table 3:** microRNAs involved in HIF-mediated autophagy regulation in cancer.

**MicroRNA**	**Chemical Name**	**Cellular Model**	**HIF-Mediated Mechanism of Action**	**Autophagy Condition**	**References**
miR-21	microRNA-21	Cancer cell lines	Upregulation of HIF-1α	Enhanced autophagy	[106]
miR-210	microRNA-210	Hypoxic cells	Direct targeting of HIF-1α mRNA	Enhanced autophagy	[107]
miR-155	microRNA-155	Various	Inhibition of HIF-1α translation	Suppressed autophagy	[108]
miR-31	microRNA-31	Breast cancer cells	Regulation of HIF-1α stability	Enhanced autophagy	[109]
miR-29a	microRNA-29a	Lung cancer cells	Modulation of HIF-1α expression	Suppressed autophagy	[110]
miR-23a	microRNA-23a	Renal cell carcinoma	Inhibition of HIF-1α pathway	Suppressed autophagy	[111]
miR-519c	microRNA-519c	Gastric cancer cells	Targeting HIF-1α	Suppressed autophagy	[112]
miR-20a	microRNA-20a	Colon cancer cells	Downregulation of HIF-1α	Enhanced autophagy	[113]
miR-424	microRNA-424	Breast cancer cells	Regulation of HIF-1α expression	Suppressed autophagy	[114]
miR-27a	microRNA-27a	Pancreatic cancer	Modulation of HIF-1α activity	Enhanced autophagy	[115]

### 4.4. Nanoparticle-Mediated HIF-Induced Autophagy Regulation in Cancer

Recent studies have indicated that nanoparticles can effectively modulate HIF-mediated autophagy, presenting a novel therapeutic approach for cancer treatment. This review examines different nanoparticles that affect HIF-mediated autophagy regulation in cancer cells, focusing on their composition, mechanisms of action, and efficacy, as presented in Table 4. Gold nanoparticles (AuNPs) have attracted considerable interest owing to their biocompatibility, straightforward functionalization, and capacity to induce oxidative stress in tumor cells. Research indicates that AuNPs can decrease HIF-1α expression, facilitating autophagic degradation in cancer cells. In vitro concentrations of 10–50 µg/mL and in vivo doses of 10 to 30 mg/kg have demonstrated efficacy in regulating autophagy [116]. The action of liposomes in modulating HIF-mediated autophagy entails a decrease in HIF-1α levels, subsequently initiating autophagic flux and resulting in the death of cancer cells. Liposome formulations at concentrations of 1 to 10 µg/mL (in vitro) and doses of 5 to 20 mg/kg (in vivo) demonstrate significant effects on the regulation of autophagy [117]. Polymeric nanoparticles, including those composed of polylactic-co-glycolic acid (PLGA), are capable of suppressing HIF-1α expression, thereby enhancing autophagic flux. Nanoparticles have demonstrated the ability to enhance the degradation of HIF-1α, thereby increasing autophagic activity in cancer cells. Therapeutic effects are typically observed at in vitro concentrations ranging from 20 to 100 µg/mL and in vivo doses between 5 and 25 mg/kg [78]. Silver nanoparticles (AgNPs) influence HIF-1α levels, resulting in the promotion of autophagy in tumor cells. Effectiveness has been demonstrated in lung and breast cancer models, with in vitro concentrations of 10–50 µg/mL and in vivo doses ranging from 5 to 15 mg/kg exhibiting significant therapeutic effects [118]. Magnetic nanoparticles (MNPs) have demonstrated the ability to modulate HIF-1α activity and affect autophagy in liver and pancreatic cancers. MNPs enhance autophagic processes through a reduction in HIF-1α expression. Nanoparticles generally demonstrate therapeutic effectiveness at concentrations of 1–5 µg/mL in vitro and 5–10 mg/kg in vivo [119]. Carbon nanotubes (CNTs) inhibit HIF-1α expression to promote autophagic cell death. They promote the generation of reactive oxygen species (ROS), thereby activating autophagy. CNT-based treatments generally exhibit effectiveness at in vitro concentrations of 0.1 to 10 µg/mL, while in vivo doses of 5 to 20 mg/kg are considered most effective [120]. Dendrimers demonstrate the capacity to lower HIF-1α levels and stimulate autophagy in cancer cells, thereby facilitating autophagic cell death. Therapeutic efficacy is typically assessed using in vitro concentrations of 1–10 µg/mL and in vivo doses of 10 to 30 mg/kg [121]. Mesoporous silica nanoparticles (MSNs) demonstrate potential in treating breast and pancreatic cancers by regulating HIF-1α activity and facilitating autophagic clearance. MSNs promote autophagy through the downregulation of HIF-1α expression, thereby facilitating cancer cell apoptosis. In vitro concentrations ranging from 5 to 50 µg/mL and in vivo doses between 10 and 40 mg/kg effectively induce autophagy in tumor cells [122]. Gold–silver alloy nanoparticles (Au–Ag NPs) have been utilized in the treatment of colorectal and lung cancers, where they influence HIF-1α expression and promote autophagy. The photothermal characteristics of Au–Ag nanoparticles enhance their therapeutic potential, facilitating targeted treatment. Effective concentrations vary between 5 and 50 µg/mL in vitro and 10 to 30 mg/kg in vivo [123]. Quantum dots (QDs) have demonstrated the ability to inhibit HIF-1α expression in breast and prostate cancers, thereby promoting autophagic cell death. Quantum dots enhance the monitoring of drug delivery to tumor locations. Concentrations ranging from 0.1 to 1 µg/mL in vitro and doses of 5 to 15 mg/kg in vivo have demonstrated efficacy in modulating HIF-mediated autophagy [124].

## 5. Future Perspectives and Challenges of Hypoxia-Inducible Factor-Induced Autophagy Regulation in Cancer

While targeting HIF-mediated autophagy holds significant promise for cancer therapy, several challenges remain to be addressed. Knowledge of the fundamental molecular pathways is required to fully understand the complex interaction between HIFs and autophagy in the tumor microenvironment [4]. It is crucial to create selective and effective inhibitors that specifically target HIFs or critical elements of the autophagy pathway. This focused strategy can reduce off-target effects and enhance the overall effectiveness of therapeutic interventions. An enhanced comprehension of the complex molecular pathways governing HIF-induced autophagy will provide significant insights into the mechanisms that drive cancer cell survival and progression. Identifying these pathways may facilitate the development of innovative therapeutic strategies that inhibit the adaptive autophagic response in tumor cells and enhance their sensitivity to conventional therapies, potentially resulting in more effective cancer treatments with reduced adverse effects [125]. Targeting the HIF-induced autophagy axis has the potential to generate new therapeutic techniques that disrupt cancer cell survival pathways and make tumors more sensitive to traditional treatments [126]. Furthermore, the identification of small molecules or inhibitors that selectively modulate HIF-mediated autophagy holds great potential for developing targeted therapies tailored to specific cancer subtypes or patient profiles, ultimately enabling more personalized and effective treatment strategies. These compounds could also be used to overcome resistance to existing therapies by restoring proper autophagic regulation in tumor cells.

Regardless of notable advancements, numerous challenges persist in the regulation of HIF-induced autophagy in cancer. The HIF signaling pathway is highly complex, involving multiple isoforms (HIF-1α, HIF-2α, and HIF-3α), each playing a distinct role in tumor progression and autophagy regulation. These isoforms demonstrate distinct tissue-specific expression, varying stability in different oxygen environments, and unique interactions with other cellular pathways, complicating effective targeting efforts. The redundancy and overlap in the functions of these isoforms may impede the formulation of targeted therapeutic strategies for modulating autophagy in cancer cells [127]. Understanding the precise role of each HIF isoform in regulating autophagy dynamics in the tumor microenvironment is essential for developing targeted treatments with limited unintended effects. It is crucial to identify reliable biomarkers for the effective monitoring of autophagy levels induced by HIFs in cancer patients. These biomarkers may offer significant insights into the tumor microenvironment, facilitating the evaluation of therapeutic efficacy for treatments aimed at HIF-mediated autophagy and supporting more personalized and precise approaches in cancer therapy [128]. Although there are difficulties with ongoing research endeavors, understanding the molecular mechanisms underlying HIF-induced autophagy regulation has significant potential for enhancing patient outcomes and surmounting treatment resistance in cancer [10]. Addressing these challenges and adopting emerging strategies, including precision medicine, genetic profiling, and innovative therapeutic approaches, can lead to more effective and personalized treatments for cancer patients, thereby enhancing outcomes and reducing side effects.

## 6. Conclusions

The relationship between hypoxia-inducible factors and autophagy presents a potential avenue for improving cancer therapy. HIFs are essential in the regulation of autophagy pathways, offering significant potential for therapeutic interventions [4,12]. Understanding the HIF-mediated regulation of autophagy presents significant potential for addressing treatment resistance and enhancing patient outcomes in diverse cancer types. This strategy may facilitate the creation of new treatments that improve the efficacy of current therapies and tackle drug resistance. The identification of distinct HIF isoforms and their specific effectors in autophagy regulation may facilitate the development of more personalized therapeutic strategies. Additional research is required to elucidate the intricate relationship between HIFs and autophagy within the tumor microenvironment, as well as to address the challenges associated with implementing HIF-targeted therapies in clinical practice. In summary, integrating HIF biology with autophagy regulation presents considerable potential for enhancing cancer treatment.

## Figures and Tables

**Table 4:** Different types of nanoparticles, their composition, and their involvement in HIF-mediated autophagy regulation in cancer cells.

**Nanoparticle Type**	**Nanoparticle Composition**	**Cancer Type**	**Mechanism of Action**	**HIF-Mediated Autophagy Regulation**	**Concentration/** **Dose (In Vitro/In Vivo)**	**Ref.**
Gold nanoparticles (AuNPs)	Gold	Breast cancer, lung cancer	Oxidative stress induction and activation of autophagy via ROS generation	Modulates HIF-1α activity and autophagy induction	10–50 µg/mL (in vitro), 10–30 mg/kg (in vivo)	[116]
Liposomes	Lipid bilayer (phospholipids)	Glioblastoma, prostate cancer	Delivery of drugs and enhanced uptake by tumor cells	Reduces HIF-1α levels and promotes autophagic degradation	1–10 µg/mL (in vitro), 5–20 mg/kg (in vivo)	[117]
Polymeric nanoparticles	Polylactic-co-glycolic acid (PLGA)	Colon cancer, melanoma	Slow release of therapeutic agents and targeted delivery	Suppresses HIF-1α expression and enhances autophagic flux	20–100 µg/mL (in vitro), 5–25 mg/kg (in vivo)	[78]
Silver nanoparticles (AgNPs)	Silver	Lung cancer, breast cancer	Induction of apoptosis and autophagy via ROS generation	Enhances HIF-1α degradation and upregulates autophagy in cancer cells	10–50 µg/mL (in vitro), 5–15 mg/kg (in vivo)	[118]
Magnetic nanoparticles (MNPs)	Iron oxide	Liver cancer, pancreatic cancer	Magnetic targeting for tumor site-specific drug delivery	Modulates HIF-1α activity and influences autophagic processes	1–5 µg/mL (in vitro), 5–10 mg/kg (in vivo)	[119]
Carbon nanotubes (CNTs)	Carbon	Cervical cancer, leukemia	Facilitation of drug delivery and ROS generation	Inhibits HIF-1α and induces autophagic cell death in tumor cells	0.1–10 µg/mL (in vitro), 5–20 mg/kg (in vivo)	[120]
Dendrimers	Poly(amidoamine) (PAMAM)	Ovarian cancer, brain tumor	Targeted drug delivery and cell uptake enhancement	Reduces HIF-1α levels and promotes autophagic cell death	1–10 µg/mL (in vitro), 10–30 mg/kg (in vivo)	[121]
Mesoporous silica nanoparticles (MSNs)	Silica	Breast cancer, pancreatic cancer	Drug encapsulation and controlled release	Enhances autophagy through HIF-1α suppression	5–50 µg/mL (in vitro), 10–40 mg/kg (in vivo)	[122]
Gold–silver alloy nanoparticles (Au-Ag NPs)	Gold–silver alloy	Colorectal cancer, lung cancer	Combination therapy with photothermal properties	Inhibits HIF-1α and enhances autophagic clearance	5–50 µg/mL (in vitro), 10–30 mg/kg (in vivo)	[123]
Quantum dots (QDs)	Semiconductor materials (CdSe)	Breast cancer, prostate cancer	Fluorescence for tracking drug delivery	Inhibits HIF-1α and accelerates autophagy-mediated cell death	0.1–1 µg/mL (in vitro), 5–15 mg/kg (in vivo)	[124]
